# Successful treatment of a habitual patellar dislocation after a total knee arthroplasty with a closing-wedge distal femoral varus osteotomy and medial patello-femoral ligament reconstruction

**DOI:** 10.1186/s40634-020-00281-3

**Published:** 2020-09-01

**Authors:** Hidetomo Saito, Kimio Saito, Yoichi Shimada, Toshiaki Yamamura, Takahiro Sato, Koji Nozaka, Hiroaki Kijima, Masashi Fujii, Tetsuya Kawano, Naohisa Miyakoshi

**Affiliations:** 1 Department of Orthopedic Surgery, Akita University Graduate School of Medicine, Hondo 1-1-1, Akita, 010-8543 Japan; 2 Akita Sports Arthroscopy and Knee Group, Akita, Japan; 3 Sapporo Sports Clinic, Chuo-ku Kita-1-jo, Nishi-2-Tyome, Sapporo, 060-0001 Japan

**Keywords:** Mal-alignment, Revision arthroplasty, Anterior knee pain, Patella instability

## Abstract

**Abstract:**

A 68-year-old female suffering from habitual patellar dislocations following a mal-positioned total knee arthroplasty (TKA) was successfully treated with a biplanar closed wedge distal femoral osteotomy (CWDFO) and medial patello-femoral ligament (MPFL) reconstruction. To the best of our knowledge, no such case has been previously described. Our experience with this case suggests that treatment for a patella dislocation following valgus mal-positioning of TKA should be considered positively.

**Level of evidence:**

V

## Background

Patellar instability is often the cause of TKA failure [13]. The reasons underlying this instability must be determined precisely before it can be treated, including possible errors in the prosthesis implantation (such as an abnormal internally rotated femoral and/or tibial component, a poorly restored patellar offset, medialization of the femoral component or abnormal patellar resurfacing), alignment defects, an excessively large tibial tuberosity to the trochlear groove (TT- TG) distance, and/or patella alta) [11]. Among the various surgical options for treating patellar instability, the optimal procedure should be selected in accordance with the primary surgical error and secondary pathology. To our best knowledge, no successful examples of combining MPFL reconstruction and CWDFO to realign the patella following a mal-positioned TKA have been described.

## Case presentation

**Fig. 1:**
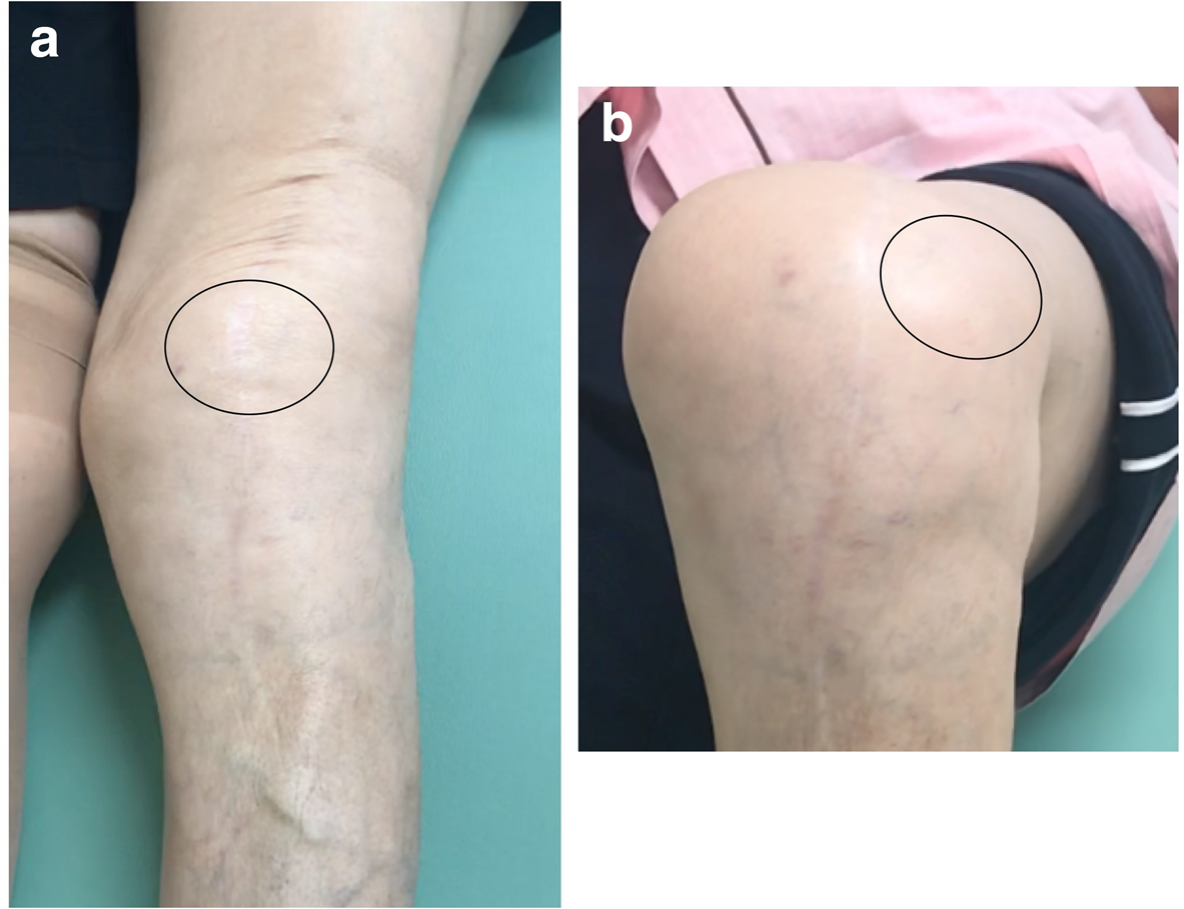
Macroscopic photographs of the clinical findings in the study patient. **a** Patella reduced in the extended knee. **b** Patella dislocated laterally in the flexed knee

**Fig. 2:**
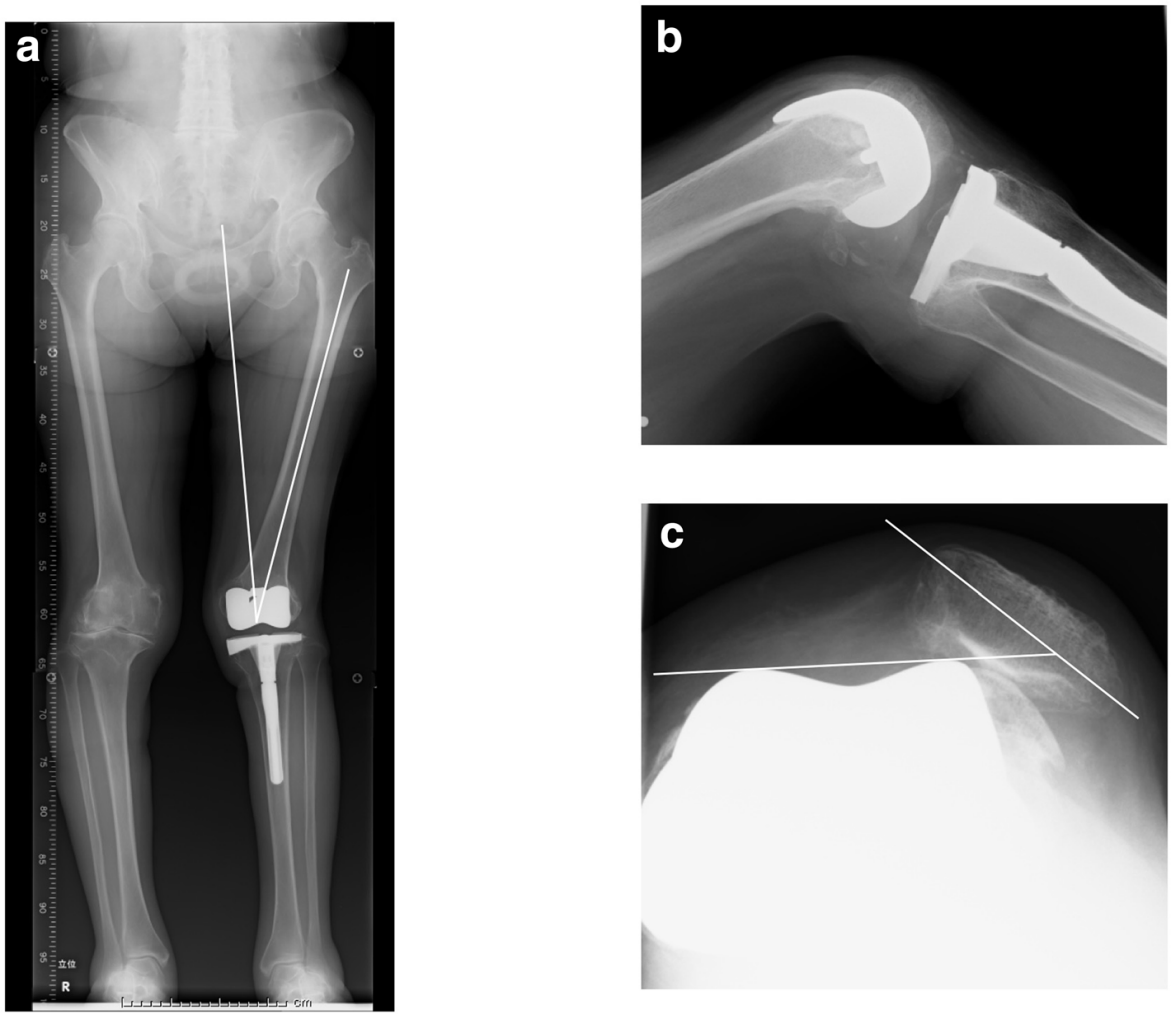
X-rays of the patient’s knee at admission. **a** Mal-positioning of the femoral component by as much as 18 degrees was evident in a long leg standing X-ray. **b** A tibial posterior sag. **c** A lateral dislocation of the patella. The patella tilt was 43 degrees

**Fig. 3:**
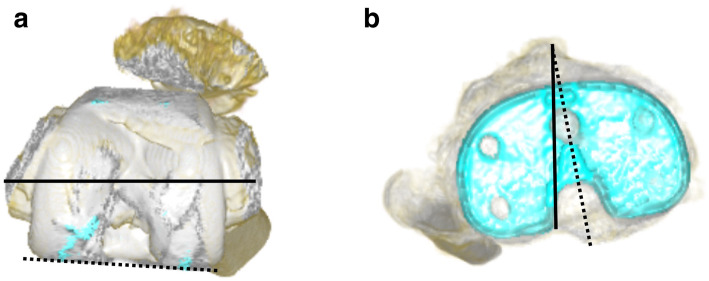
Computed tomography analysis of the patient. **a** Femoral component installed in the proper position. Solid line represented transepicondylar line, broken line represented posterior femoral condylar line. **b** Tibial component installed in a position of mild external rotation rather than internal rotation. Solid line represented Akagi’s line, broken line represented antero-posterior axis of tibial prosthesis

A 68-year-old female was referred to our hospital due to habitual patellar instability. She had received a TKA without patella resurfacing at another hospital 15 years previously but had been suffering from severe knee pain and patella instability for 8 years. She walked lame due to pain. During her physical examination, the patella was found to have dislocated laterally towards the knee flexion and reduced the normal position to 30 degrees flexion during extension (Fig. 1). The results of an apprehension test and posterior drawer test were positive and the knee was significantly laxed under both valgus and varus stress testing. An artificial mass was palpated at the medial side of the distal femur. X-rays of the knee revealed a tibial posterior sag, the femoral component had been installed at an extremely valgus position, and the patella surface was still native, but irregular and sclerotic. No implant loosening was evident (Fig. 2). The FTA was measured at 159°, HKA at 9° and mLDFA at 80°, all of which were out of the normal range. CT analysis of the axial section confirmed that the femoral and tibial prosthesis had been adequately installed (Fig. 3). TT-TG distance was 8 mm. We thus concluded that the habitual dislocation and polyethylene insert post failure were due to valgus mal-installation of the femoral component and a lack of insert thickness during the initial TKA despite proper rotational installation.

**Fig. 4:**
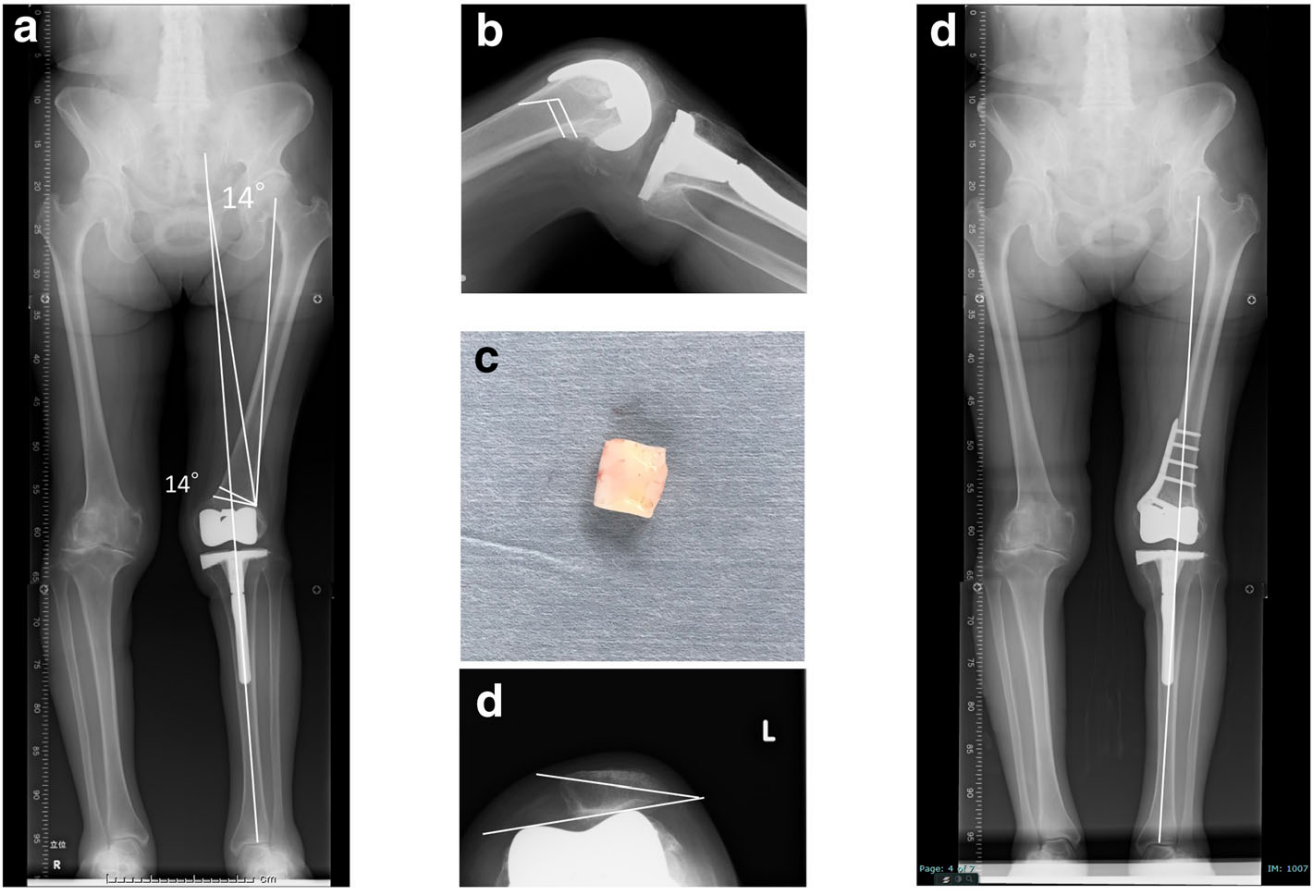
X-rays after combined CWHTO with medial plication and lateral release. **a** Preoperative planning using Miniaci method. **b** Preoperative planning of biplanar CWHTO in lateral view. **c** Broken polyethylene post was exposed from medial aspect of the knee with a stab incision. **d** An eighteen degrees of patella tilt still remained after CWDFO. **e** Postoperative long leg standing X-ray

**Fig. 5:**
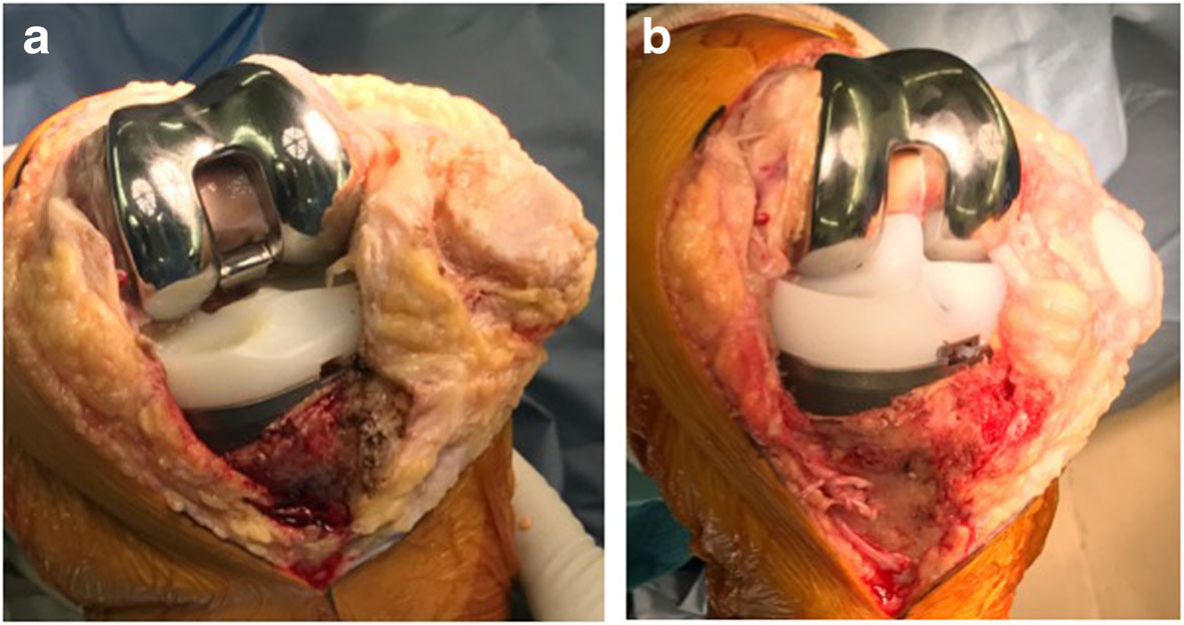
Intraoperative macrographs. **a** the post of polyethylene insert was broken, and the patellar chondral surface was irregular and erosive. **b** the broken insert was replaced by thicker one to restore the varus-valgus instability the patella was resurfaced

We conducted step wise surgery to preserve the prosthesis and avoid the bone loss and complications related to prosthesis removal. In the first step, we planned a varus biplanar CWDFO exposing the broken region of the polyethylene insert, and achieve medial plication and lateral release. According to Miniaci method [7], we performed a 14 degrees of the biplanar varus medial CWHTO to achieve the weight bearing line passing through the center of the knee (Fig. 4a, b). The post of polyethylene insert was removed from fat tissues at medial part of distal femur (Fig. 4c). Although medial plication and lateral release after CWDFO, the patella still remained subluxated (Fig. 4d). Postoperative long leg standing X-ray revealed the weight bearing line almost passed through the center of the knee (Fig. 4e). Because the patella instability and femoro-tibial instability due to a lack of insert thickness were sustained, in the second surgery, we thus conducted patella resurfacing and an replaced a 14 mm thickness of polyethylene insert to 20 mm thickness (Fig. 5a, b). Subsequent MPFL reconstruction was then conducted to normalize the lateral subluxated patella tracking. Regarding MPFL reconstruction, a single-tailed hamstring tendon graft was fixed in 30° - 45° of knee flexion after the graft was placed both in Schöttle’s point [10] of the femur using ACL TightRope® (Arthrex, Inc. Naples, FL, US) and oblique single tunnel in patella.

**Fig. 6:**
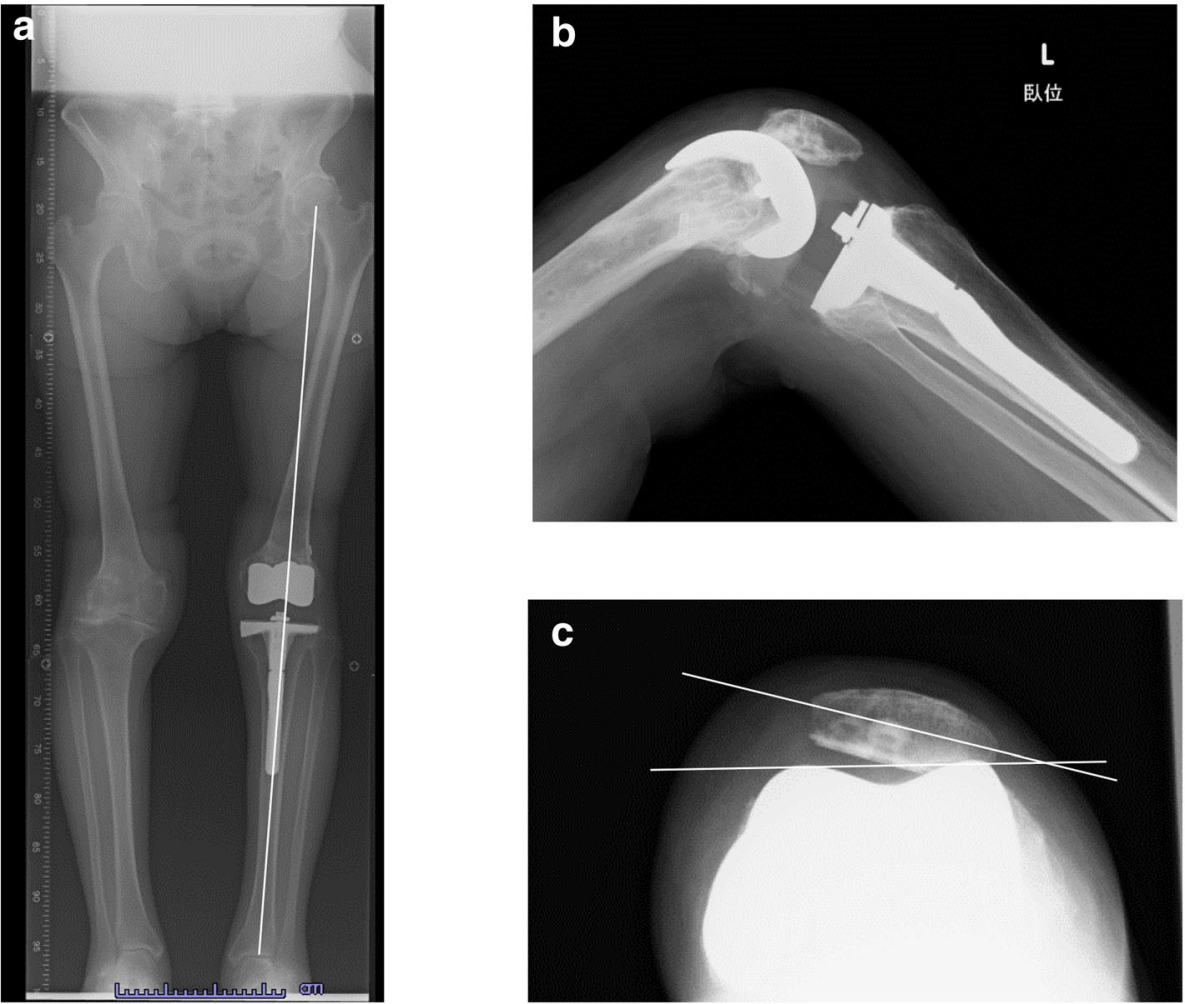
X-rays at the final follow-up. **a** Normalized alignment of the left lower extremity. **b** Disappearance of the posterior tibial sag. **c** Patella reduced but patella tilt remained as much as 15 degrees

At the 24-month postoperative follow-up, the patient was able to use stairs without any support. The active range of motion (ROM) was now an extension to 0° and flexion to 130° without extension lag. Physical examinations revealed that the patella was stable in a lateral stress test with no apprehension signs. X-rays at 24 months after her second corrective surgery demonstrated good congruence of the patello-femoral joint (the tilt angle improved from 40° to 20°; the bisect offset improved from 121 to 86%, and finally to 60%), the disappearance of any tibial posterior sag, normal alignment of the lower extremity with an FTA of 172° and a HKA of 2° (Fig. 6). There was no further patellar dislocation. The Kujala functional score and the Oxford knee score improved from 24 to 58, and from 28 to 40, respectively.

## Discussion

**Fig. 7:**
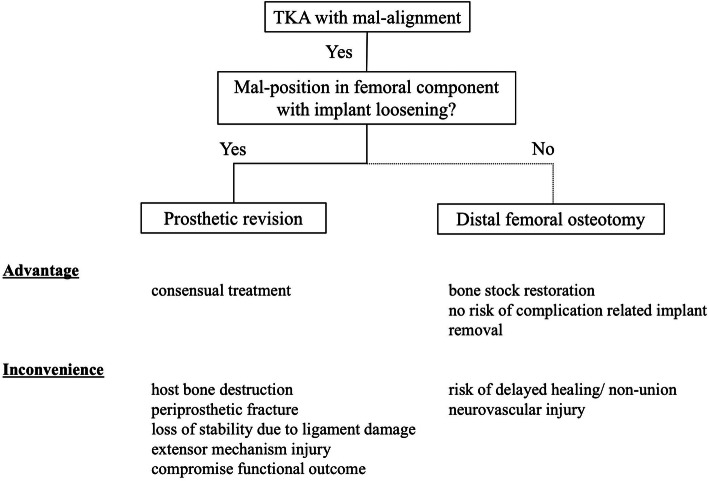
Surgical strategy, their advantage and inconvenience between osteotomy and prosthetic revision

We showed for the first time that a combination of CWHTO and an MPFL reconstruction can treat a habitual patella dislocation caused by a mal-positioned TKA with excellent clinical outcomes at 2 years follow-up. Despite a decrease in the frequency of patella instability due to improvements in implant design and surgical technique, this adverse event still accounts for about 10% of all TKA complications [6]. Multiple technical factors are known to affect patellofemoral instability after TKA, including internal rotation of the femoral or tibial components, lateral setting of the patellar component, under-resection of the patella, and the use of an oversized femoral component [1, 3]. Lateral retinacular release with vastus medialis plication, MPFL reconstruction, distal realignment procedures, or revision arthroplasty have been recommended to treat this complication, depending on the etiology [1, 3, 5]. Revision arthroplasty is justified when the patellar instability is attributable to component mal-positioning [4]. In cases of mal-positioning, a revision arthroplasty has been usually conducted to adequately fix the implant whilst minimizing bone loss and preserving bone quality, despite the negative effects on the long-term integrity of the primary TKA. On the other hand, as patellar instability in association with genu valgum has been described [9], we hypothesized the reason why the patella in the current case has been gradually dislocated was due to the significant valgus alignment. Because successful CWDFO combined with medial reefing and lateral release in cases of habitual or recurrent patellar dislocation with genu valgum was reported [8], CWDFO should therefore be considered a viable surgical option for a habitual patellar dislocation with genu valgum created by a mal-positioning TKA, despite osteotomy was not the consensual treatment in case of TKA with malalignment. Moreover, from the perspective of the stability between the femur and the mal-positioned prosthesis, the presence of implant loosening was considered to be very important in determining surgical procedure in terms of minimize the surgical complication [2]. Because implant removal could potentially significantly increase the complexity of the revision, extend the length of the surgical procedure and subsequently increase the risk of infection and could further compromise the ultimate functional outcome of the patient. Therefore, we proposed the surgical strategy, advantage and inconvenient for both techniques between prosthetic revision and distal femoral osteotomy (Fig. 7). Above all, we have now successfully performed an alignment correction using a combination of CWDFO and MPFL reconstruction.

As to our management of the patella resurfacing, problems in this knee were patella instability and patello-femoral osteoarthritis (OA) induced by mal-aligned primary TKA without resurfacing patella which was conducted 15 years ago. As femoral mal-aligned prosthesis position caused overloading of the patellofemoral joint which create osteoarthritis [12], radiographs in the current case revealed patella surface irregular and sclerotic. Thus, we thought both the patella stability could be regained by DFO and MPFL reconstruction, and patella OA was resolved by resurfacing in the second stage to minimize complications with multiple surgeries. The results in our current case were excellent and the speed of functional recovery of her knee was highly satisfactory.

We conclude from our present case report that to obtain the desired clinical outcomes when correcting a patella instability after a mal-positioning TKA, in special cases it seems to worthwhile to focus on preserving the prosthesis via an osteotomy and to therefore realign the patella instead of replacing it.

## Abbreviations

TKA: Total knee arthroplasty
MPFL: Medial patello-femoral ligament
CWDFO: Closing-wedge distal femoral osteotomy
FTA: Femoro-tibial angle
HKA: Hip-knee-ankle
mLDFA: Mechanical lateral distal femoral angle
CT: Computed tomography
ROM: Range of motion
